# Frequency shifting approach towards textual transcription of heartbeat sounds

**DOI:** 10.1186/1480-9222-13-7

**Published:** 2011-10-04

**Authors:** Farshad Arvin, Shyamala Doraisamy, Ehsan Safar Khorasani

**Affiliations:** 1 Department of Computer Engineering, Science and Research Branch, Islamic Azad University, Tehran, Iran; 2 Department of Multimedia, Faculty of Computer Science & IT, University Putra Malaysia, Serdang, Selangor, Malaysia; 3 Department of Software Engineering, Mashhad Branch, Islamic Azad University, Mashhad, Iran

## Abstract

Auscultation is an approach for diagnosing many cardiovascular problems. Automatic analysis of heartbeat sounds and extraction of its audio features can assist physicians towards diagnosing diseases. Textual transcription allows recording a continuous heart sound stream using a text format which can be stored in very small memory in comparison with other audio formats. In addition, a text-based data allows applying indexing and searching techniques to access to the critical events. Hence, the transcribed heartbeat sounds provides useful information to monitor the behavior of a patient for the long duration of time. This paper proposes a frequency shifting method in order to improve the performance of the transcription. The main objective of this study is to transfer the heartbeat sounds to the music domain. The proposed technique is tested with 100 samples which were recorded from different heart diseases categories. The observed results show that, the proposed shifting method significantly improves the performance of the transcription.

## Introduction

Auscultation is the most remarkable approach that has been used in diagnosing many cardiovascular diseases for many years. It still plays an important role in the diagnosis of heart disease. Sounds produced by the heart frequently reflect the structural abnormalities of the heart. Physicians use the stethoscope as a common tool to listen to the heart sounds and make a correct diagnosis. Modern stethoscopes are making the auscultation easier to be done. Despite murmurs and tones are easily distinguished, weak murmurs and below audibility threshold easily disappear in background sound. Analysis of heart sounds and extraction of its audio features would be important towards the development of automatic diagnosis systems. Phonocardiogram (PCG) is a diagram of sonic vibration of heart beats. Most researches used PCG as an audio input of system to apply different techniques of digital signal processing [1-3]. Based on characteristics of the audio signals, it is possible to apply various signal processing and modeling approaches. Healthy heart sound includes symmetric cycles and pulse values. In contrary, unhealthy heart sounds are commonly disordered by different unexpected frequencies.

Segmentation is a technique for separating cycles and its pulses [2,3]. Classification of heart sound is another research area that divides heartbeat sounds in different clusters based on their characteristics [1,4,5]. In the similar study, neural network has been used for classification of different heart sounds such as normal, systolic and diastolic murmurs [6]. A high performance localization technique of the first heart sound pulse was proposed in [7]. The localization was performed based on an additional enhancement to improve the accuracy of pulse detection. In our previous study on real-time segmentation [8], a simple segmentation technique using amplitude reconstruction was proposed which divided the heartbeat sound pulses with a high accuracy. However, the limitation was to lose of low-amplitude harmonics.

Automatic music transcription [9-12] is an approach to process the audio signals to extract the pitch levels that can be notated as musical notes and the music. Most researches in automatic music transcription attempted to increase the accuracy of the transcription to cover different frequency levels [9,11]. Transcription can be applied on heartbeat sounds in order to represent heartbeat sounds with the music notation. In previous studies [13-15], heart sounds were represented with MIDI (Musical Instrument Digital Interface) format. A good performance of transcription was illustrated in those studies. For long duration sampling of the heartbeat sounds and developing a biomedical database, text-based formats (i.e. MIDI) are the suitable mediums to convert and store the biomedical signals. Text-based music information retrieval [16,17] allows developing query-based system to highlight various events of heartbeat sounds in particularly. In our previous study [18], music transcription of heartbeat sounds was performed that demonstrated good accuracy for different heart sound samples. We proposed several preparation techniques for de-noising and cleaning heart sound signals in order to use in real-time systems. The results showed that, heart sounds can be represented as musical notation. Since heart sound signals are in very low-frequency domain [19], automatic transcription techniques that are used for music transcription are not suitable for this particular application. Therefore, in order to provide a high accuracy transcription, two methods can be used. The first method is to provide an automatic transcription technique with a new configuration to cover very low-frequency spectrum which requires complex algorithms and several modifications. The second method is to transfer the heartbeat sounds to the frequency that is used by music instruments, which allows utilizing the ordinary music processing methods.

In this paper, we propose a frequency shifting (transferring) method to increase the accuracy of the heartbeat sounds transcription. We modify automatic music transcription methods to be used in specific frequency spectrum. The process begins with a frequency estimation technique using Fast Fourier Transform (FFT), a commonly used technique. Heart sounds are divided in several parts with similar size that is called window. Thus, FFT is applied for each window and the estimated frequency is approximated to the nearest pitch number. The main problem in this step is the lower frequency of heart sounds in comparison with music. The proposed shifting method aims to solve the problem with transferring the low-frequency samples to high-frequency notes (music instruments). Moreover, the textual transcription is implemented in two processing methods which are real-time (RT) and non-real-time (NRT). The performance of the transcription is investigated in both methods.

## Music Transcription

Automatic music transcription is a technique to analyze audio signals in order to extract the pitch levels and transcribed as musical notation. The pitch extraction or pitch tracking for monophonic music starts with note or pitch onset detection, followed by a nearest pitch approximation [10]. Monophonic music in this context is when a single note or onset is sounded at any one point in time as opposed to polyphonic whereby multiple onsets may occur at a given point in time. Pitch approximation is based on the fundamental frequency (*f*_0_) of the given piece of music to find nearest note number relative to that particular note. In order to extract the fundamental frequency of a played note, Fast Fourier Transform (FFT) would be the most appropriate method. FFT detects all the frequencies in a given window. An audio signal stream is usually broken into smaller sections called windows for analysis. The following formula illustrates the frequency distribution, *X*, from a window with size of *N *samples:

(1) $$X_{k}=\overset{N-1}{\underset{j=0}{\sum }}x_{j}e^{-i\frac{2\pi kj}{N}}\;,\;k\in \left\{0,1,\cdots \;,N-1\right\}$$

Transcription of polyphonic music is more complex than monophonic due to the occurrence of several notes at a given point in time [10].

## Heartbeat Sounds Transcription

This section presents the transcription technique that is used to process the heartbeat sounds. According to the nature of the heart sound, the music signal processing techniques can be adopted with a few modifications in terms of frequency and window sizes. These modifications are required due to differences between music and heartbeat sounds' characteristics.

### Heartbeat Sounds

**Figure 1:**
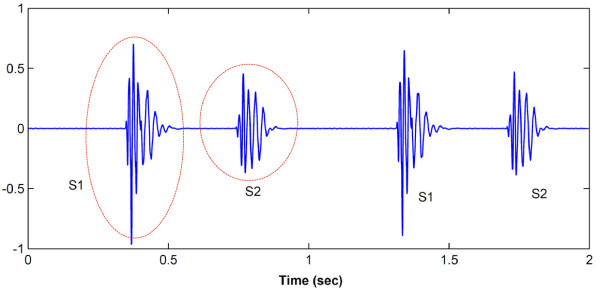
**Waveform of a normal heart sound**. S1 denotes first heart sound S2 denotes second heart sound.

Heartbeat sounds are semi-periodical signals that are generated by blood turbulence and the beating heart. These sounds provide important and common ways for diagnosing of heart diseases with its ease of availability as well as in a cost-effective way. A heartbeat sound normally consists of two pulses; first heart sound (S1) and second heart sound (S2). Figure 1 shows a normal heart (healthy) sound waveform. The duration of heart sound pulses is approximately 100 ms [3,20]. This duration is sufficient for applying signal processing techniques. Moreover, the frequency of heart sounds is low in range between 20 and 150 Hz [19]. Hence, the heart sounds can be represented in first and second music octaves.

**Figure 2:**
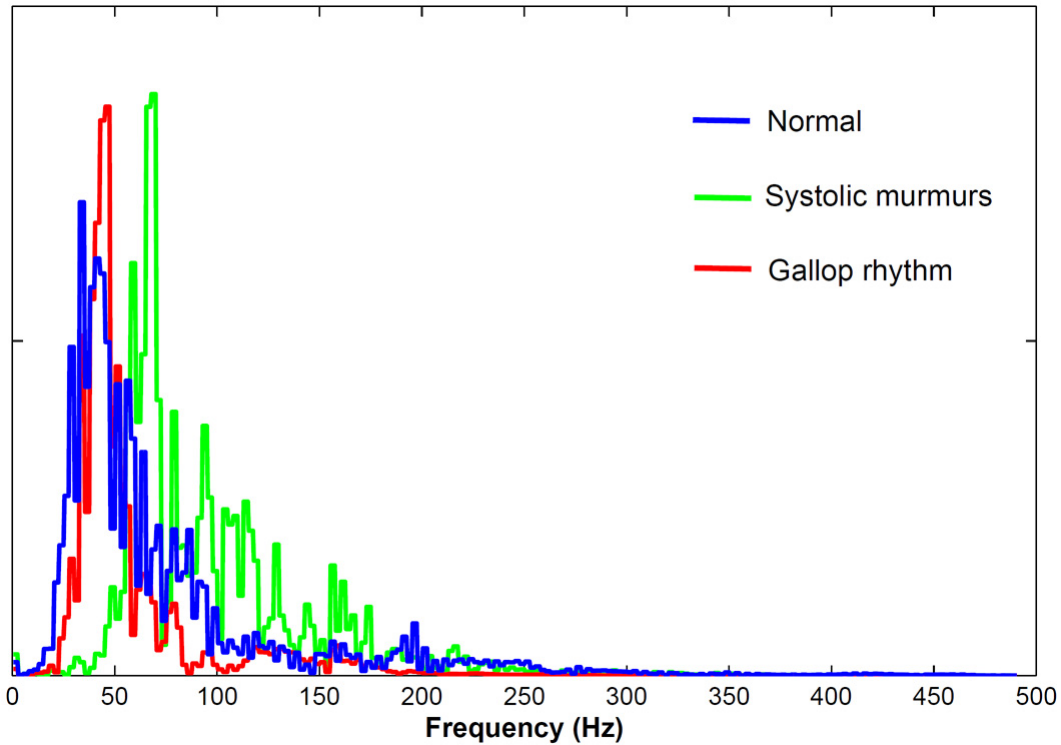
**Frequency distribution of heart sounds in different samples (normal, gallop rhythm and systolic murmurs)**.

Figure 2 shows the frequency distribution of three randomly selected samples of heart sounds from normal, gallop rhythm and systolic murmurs cases. Clearly, it has been shown that the signals are mostly in low-frequency spectrum.

### Preprocessing

**Figure 3:**
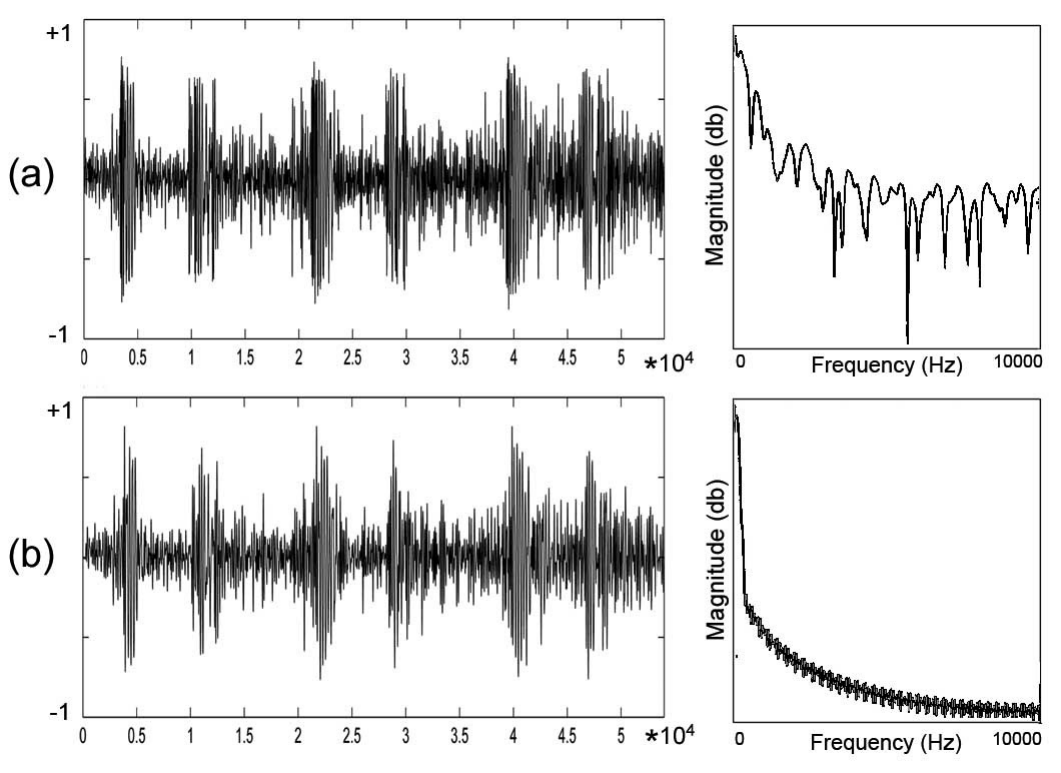
**Frequency distribution of a heartbeat sound**. (a) Before preprocessing and (b) After preprocessing.

Generally, the recorded heart sound consists of the background audio and other organs sounds. Therefore, preprocessing of heartbeat sounds is an important task for de-noising of samples [18]. Based on our domain of study, the unexpected sounds (e.g., other organs) are assumed as the noise. Before frequency estimation of the heart sound, different levels of the preparation on frequency and amplitude domains are required [8]. Figure 3 shows a randomly selected heart sound from healthy (normal) category before and after preprocessing. The frequency distribution of the first pulse is shown in the right side of the each PCG. As it is illustrated in the frequency distribution, the heart sounds consist of various higher frequencies with higher magnitudes. Hence, filtering and noise cancellation of unexpected signals are required. A low-pass filter with *f_pass _*= 250 Hz and *f_stop _*= 400 Hz is applied for heart sound samples.

### Textual Transcription

In this paper, we aim to process heart sounds with a monophonic pitch tracking method. However, some modifications must be applied for the case of onset time detection and frequency estimation of heart sounds with low frequencies. In order to simplify the process and provide outputs with similar duration, we eliminate the onset detection. Therefore, the heart sounds are processed in small size windows (with size of *w*). The results are stream of text that each byte shows binary value of the pitch numbers. On the other hand, to reduce the number of calculations frequency is limited between 1 and 500 Hz. Frequency estimation is the main step in musical transcription. After that, the relevant pitch number is estimated with a simple calculation by the following formula:

(2) $$N\left(p\right)=40\log\left(\frac{f\left(p\right)}{261.6}\right)+60,$$

where *f*(*p*) denotes the estimated frequency and *N*(*p*) is the nearest pitch approximation. The note number 60 is the musical note C_4 _with frequency value of 261.6 Hz. This formula shows that, if the value *f*(*p*) is increased to 2 times, the value of 12 which is an octave interval is added to the *N*(*p*). Each calculated section is equivalent to one musical note, and generates binary codes based on *N*(*p*).

**Figure 4:**
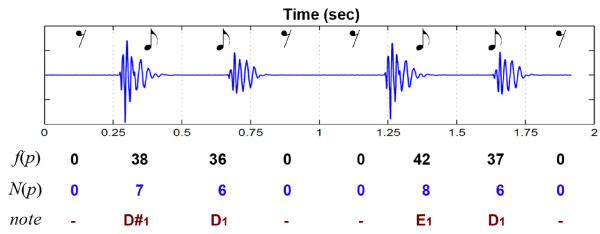
**A sample of transcription with 4 pulses using window size of 250 ms**. *f*(*p*) shows the extracted frequency, *N*(*p*) is the calculated pitch number and *note *indicates the relative music note.

Figure 4 shows an example of transcription for a normal heartbeat sounds with window size of 250 ms. In this sample, each window is converted to a eighth note duration. There is a slight correlation between pulses and frequencies which in most normal cases S1 shows higher frequency than S2.

We use textual transcription term instead of musical transcription due to storage format of the converted samples that is a plain text consists of binary value of the note numbers. Moreover, the notes are started periodically with a constant window size.

## Frequency Shifting

**Figure 5:**
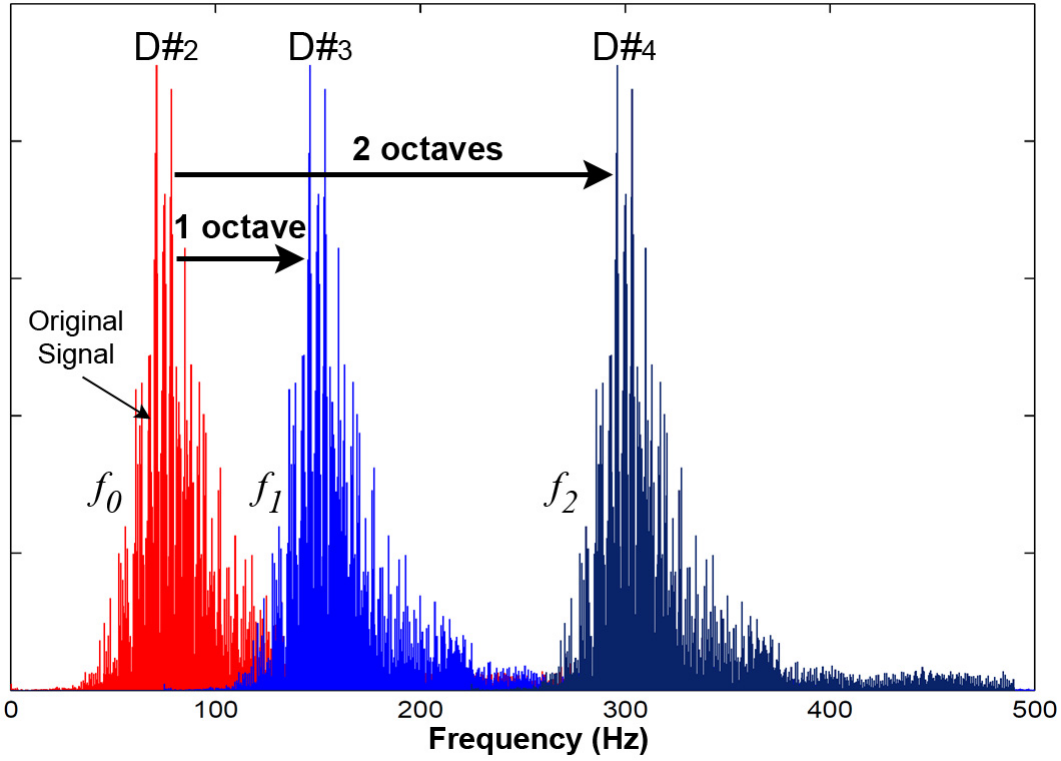
**Sample of frequency shifting for one and two octaves**. *f*_0 _is the original sample, *f*_1 _is the result of an octave shifting and *f*_2 _is the result of two octaves shifting. D^# ^is the relative note.

The preliminary experiments revealed a low accuracy of transcription due to the low frequency of heart sound [21]. Therefore, we propose a frequency shift method that increases the frequency level of the heart sound to provide an accurate transcription. Based on this, a constant shifting value, *f_sh_*, is added to the extracted frequency in order to reach a high frequency note. The shifting size impacts the performance of the transcription that must be investigated to find a suitable value. Figure 5 shows an example of frequency shifting for one and two octaves. In that example, if original signal is assumed as *f*_0 _hence the first octave shifting is performed by *f*_1 _= 2 * *f*_0 _and second octave is calculated with *f*_2 _= 2 * *f*_1 _(*f*_2 _= 4 * *f*_0_). As it is shown in Figure 5, each window consists of vast distribution of the frequencies. This distribution must be limited to achieve an accurate transcription. In a previous study, we proposed an amplitude reconstruction method that passes the high magnitude signals and reconstructs the lower magnitude than a specified threshold level [8]. Therefore, by applying that method, the low magnitude signals and frequencies (known as harmonics) are eliminated.

**Figure 6:**
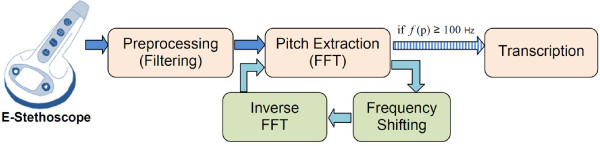
**Process diagram of transcription**. Frequency shifting is called when frequency is lower than 100 Hz.

Figure 6 shows our process diagram for heart sounds transcription. The pitch extraction function estimates the frequency of the specific window. The frequency shifting is called for the cases of lower frequencies than 100 Hz. After the shifting task, the inverse FFT is used to prepare an audio signal from the shifted sounds. In some cases with very low frequency (< 50 Hz), the shifting task must be applied at least two times to reach the accepted minimum frequency level.

## Experiments

This section explains experimental configuration and platforms that are utilized to perform the proposed transcription. In addition, the method of sampling and resources are described in this section as well. In this study, the aim is in obtaining a high accuracy transcription of the heartbeat sounds with both real-time and non-real-time methods. Furthermore, the size of the converted textual files must be small that can be used for a continuous transcription of the audio heartbeat data stream.

For investigating the accuracy of the transcription, the ratio of correct notes transcribed, *n_t_*, to the total transcribed notes, *N*, is calculated.

(3) $$P_{acc}=\frac{n_{t}}{N}\times 100$$

### Experimental Setup

The proposed transcription method is implemented using real-time and non-real-time processes. We investigate both processing methods in terms of accuracy and feasibility.

#### Real-Time Process

**Figure 7:**
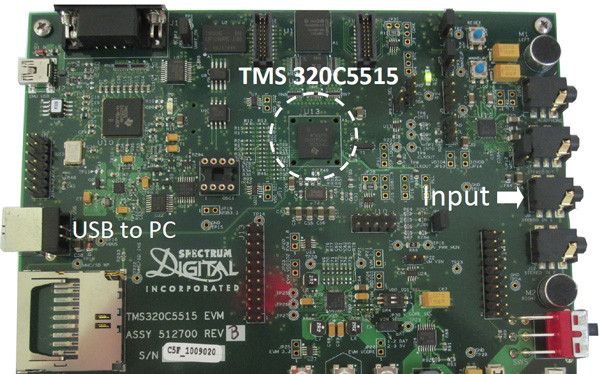
**DSP evaluation module**. The main processor is TMS320C55152 DSP.

With the real-time (RT) process, a digital signal processor (DSP) evaluation module was utilized. A TMS320C55 series DSP was deployed as the main processor. The DSP is a 16-bit processor with on-chip 320 KB memory. Figure 7 shows an image of the evaluation module. We use a single input channel of the module which directly captures the heart sounds. To accelerate the frequency calculation, TMS320C5515 was equipped with a hardware accelerator for FFT computation which estimates the frequency of the given signals simultaneously.

After real-time processing of each window, the estimated byte (pitch number) is sent to the PC to save as a plain text. The aim of the use of this module is to evaluate the possibility of the RT process for the future applications.

#### Non-Real-Time Process

For the non-real-time (NRT) process, we use MATLAB software version 6. Each record is loaded into an array of integer values where each cell contains a magnitude of a sample. The array is divided into several sub-arrays with length of window and pitch extraction is performed for each window separately. The extracted notes are then stored as plain text format. To reduce the number of calculations, the estimated frequencies are limited in a range from 0 to 500 Hz.

### Data Collection

The proposed transcription technique and the shifting method must be evaluated for both healthy and unhealthy heart sound samples. Following this, the samples are recorded from different heart problems as well as healthy heart sounds. In this study, 100 heart sounds were recorded by an electrical stethoscope (3M-Littman Stethoscope) and were checked by a cardiologist. The samples were categorized in 8 different groups based on heart diseases. Table 1 shows the number of recorded samples in different categories. In this study, we are not interested to propose an automatic heart diseases detection or classification. Although different categories have been obtained, these were grouped as healthy and unhealthy for evaluating the performance of the transcription. Therefore, the proposed method is tested with 84 records from heart diseases and 16 healthy heartbeat sounds. The duration of each sample is around 10 sec.

**Table 1:** Category of samples that are used in this study

Name	Category	Number of samples
A1	Normal Sound	16

A2	Split Second Sound	12

A3	Ejection Sound	10

A4	Clicks	12

A5	Gallop Rhythm	13

A6	Systolic Murmurs	13

A7	Diastolic Murmurs	12

A8	Continues Murmurs	12

## Results and Discussion

**Figure 8:**
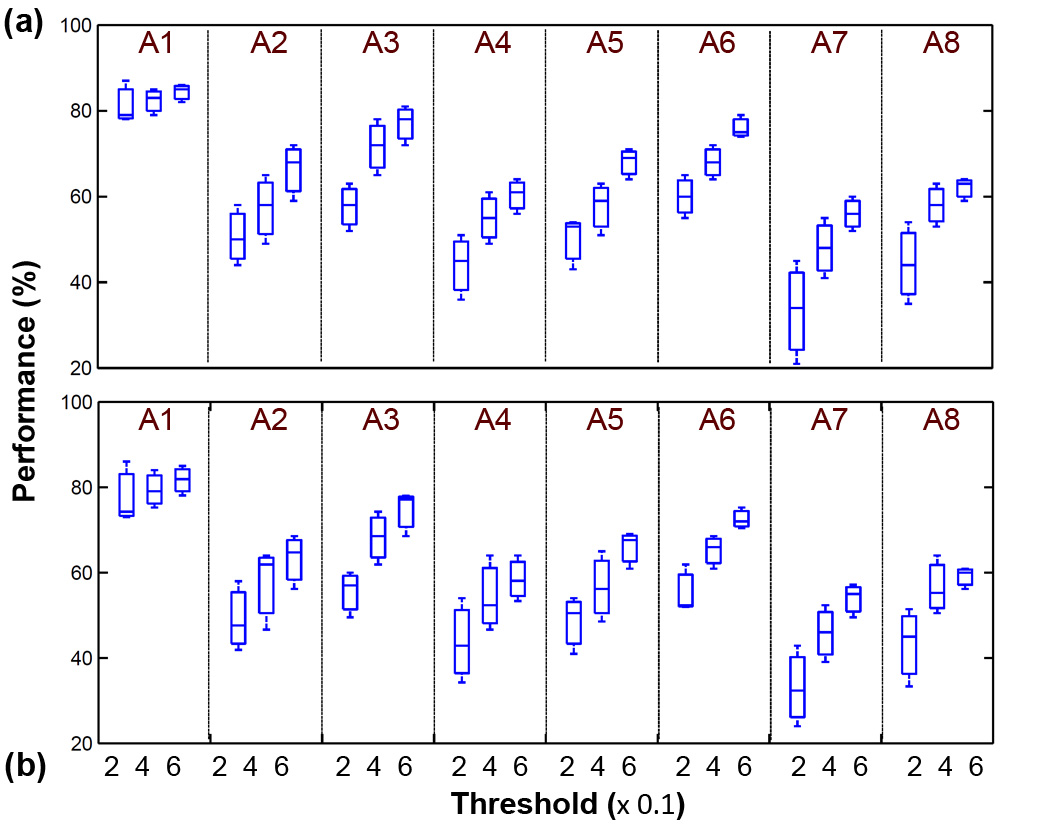
**Performance of the transcription in different threshold levels**. (a) NRT and (b) RT processes. Categories are denoted by A1 - A8.

In this section, the results of the performed experiments are discussed. The first part of our experiments estimates the appropriate threshold level in order to define an experimental framework. Figure 8 reveals the results of three different threshold levels with respect to the maximum peak that has been occurred in samples. The threshold levels are *T *∈ {0.2, 0.4, 0.6} and the window size is 250 ms. The observed results reveal that, an increase in threshold level results in an increase with the performance of the transcription for both RT and NRT processes. By increasing the threshold level, the low magnitude samples (including noise and harmonics) are eliminated and high energy samples are used for sampling. As it is expected, the NRT process has better performance in most cases in comparison with RT process.

Therefore, the higher ratio of threshold values show better performance. This results were also illustrated in our previous study [18]. In the following experiments, the threshold level was assigned as *T *= 0.6 *A_max_*, where *A_max _*denotes the sample with maximum magnitude.

**Figure 9:**
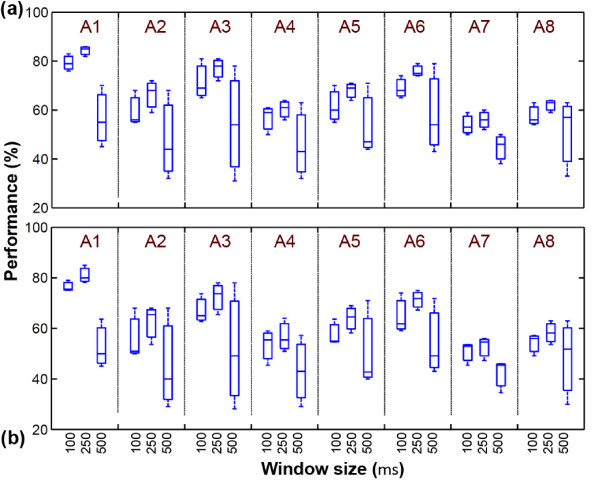
**Effects of window size in transcription of the different categories**. (a) NRT and (b) RT processes.

The second part of the experiments is to estimate a suitable size of the window (*w*) for frequency estimation process. In this regard, different window sizes are evaluated (*w *∈ {100, 250, 500} in milliseconds). Figure 9 shows the performance of the transcription for different categories with RT and NRT processes. The observed results show that, window sizes of 100 and 250 ms are suitable duration for process. However, in large size window (500 ms), some events are lost due to coverage size of the window. As an example, in some windows more than one note (pulse) may appear. Although with a short window (100 ms) the performance is good, the number of notes is extremely increases and size of the recorded text file is become larger than window with size of 250 ms. The results of the experiment show that, utilizing 250 ms as the window size gives higher performance and it provides relatively small output size.

**Figure 10:**
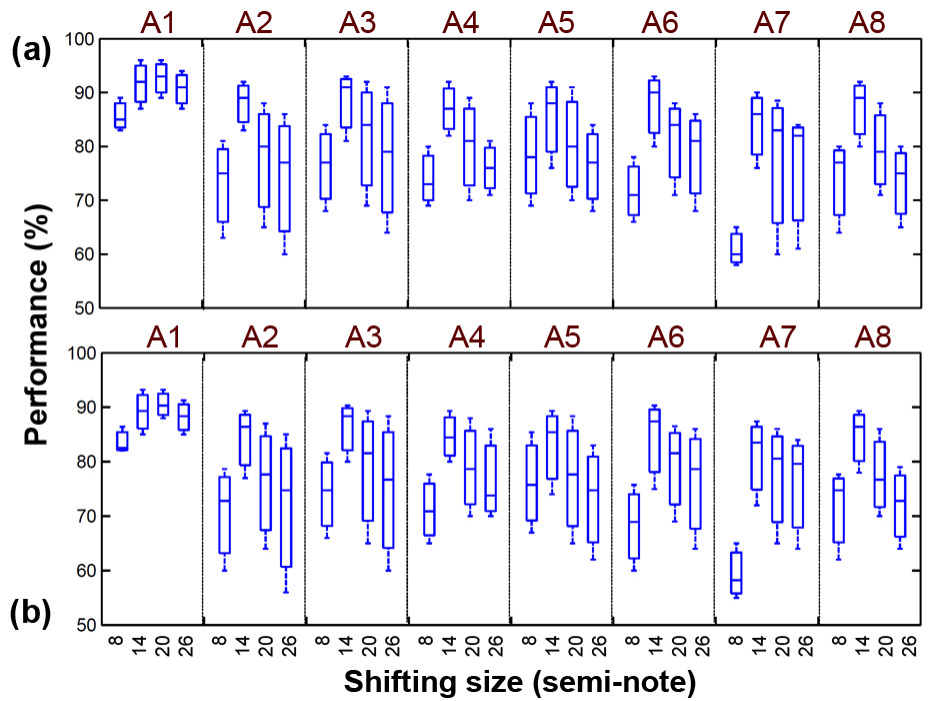
**Accuracy of the transcription in different categories as a function of different shifting sizes**.

The important part of the experiments is to investigate the effects of the different amounts of the frequency shifting in order to find a suitable shifting size. Figure 10 shows the average results of both processing methods (RT and NRT) for the shifting method with different amounts (8, 14, 20 and 26 semi-notes). This method has different impacts on our samples with respect to the categories. Totally, shifting with 14 semi-notes illustrates better performance in most cases. Increasing the shifting size more than two octaves reduces the performance due to the loss of some higher frequencies, which may occur in filtering process.

**Figure 11:**
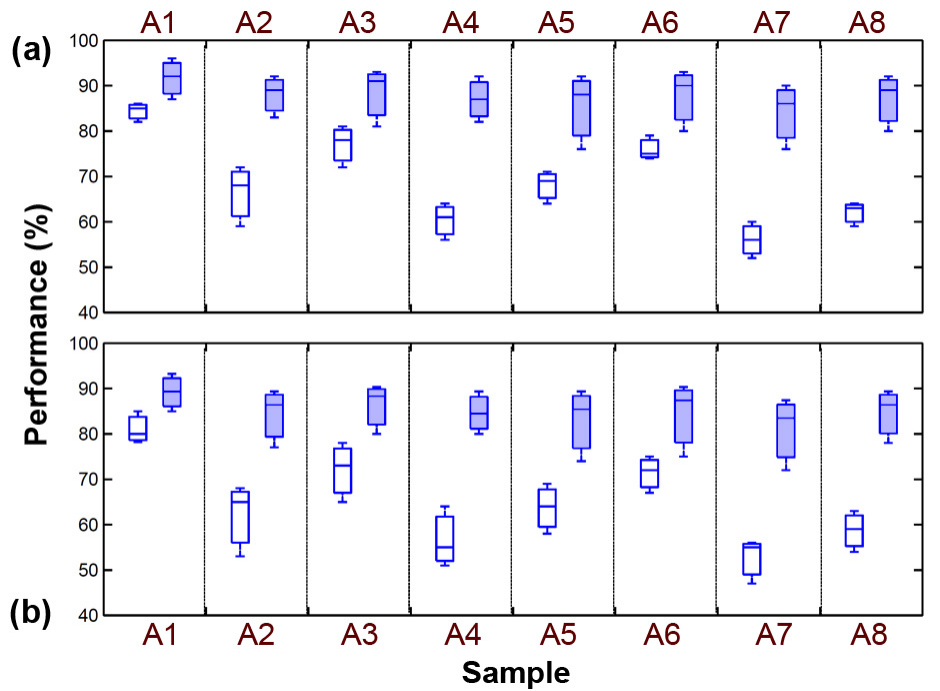
**The accuracy of the proposed transcription methods**. (a) NRT and (b) RT processes. Empty box denotes before frequency shifting and bold box denotes after frequency shifting.

Finally, an experiment is performed with the obtained configuration values (*T *= 0.6 *A_max_*, *w *= 250 ms and *f_sh _*= 14 semi-notes). Figure 11 shows the results of the performance evaluation. It shows that, the frequency shifting significantly increases the accuracy of the transcription regardless of the categories. The average accuracy of 90% and 85% for NRT and RT respectively in unhealthy cases and 95% and 89% for NRT and RT respectively in normal heart sounds are obtained. Therefore, the frequency shifting increases the accuracy of transcription that was performed in [18].

In contrast with the previous studies [13,14], the proposed frequency shifting could cover the low frequency samples without complex calculations. In addition, based on the duration of each pulse that is enough for applying the frequency estimation, with selecting small window sizes it was shown to be possible to implement the frequency shifting in real-time applications.

## Conclusion

In this paper, we proposed a frequency shifting method in order to increase the accuracy of the transcription. This method was tested in various recorded heart sounds samples including healthy and unhealthy cases that were categorized in 8 groups. The suitable values for configuration parameters of signal processing such as window size and threshold level were estimated by the initial experiments (*w *= 250 ms and *T *= 0.6). Following this, the shifting method was evaluated and an appropriate shifting size (14 semi-notes) was selected. The performance of the transcription was tested in different heart sound samples using real-time and non-real-time processes. The observed results showed that non-real-time process has a better performance in comparison with real-time process (95% and 90% for healthy and unhealthy cases respectively). The accuracy of the real-time process was also good (89% and 85% for healthy and unhealthy cases respectively). It reveals that, this method can be used in real-time systems such as house hold heart problem detection systems as early warning systems.

## Competing interests

The authors declare that they have no competing interests.

## Authors' contributions

FA carried out signal processing experiments and drafted the manuscript. SD participated in technical issues and supervised the study. ESK performed data collection and information retrieval experiments. All authors read and approved the final manuscript.

## References

[B1] Babaei S Geranmayeh A Heart sound reproduction based on neural network classification of cardiac valve disorders using wavelet transforms of PCG signals Computers in Biology and Medicine 2009 39 8 15 10.1016/j.compbiomed.2008.10.004 19081085

[B2] Sepehri A Gharehbaghi A Dutoit T Kocharian A Kiani A A novel method for pediatric heart sound segmentation without using the ECG Computer methods and programs in biomedicine 2010 99 43 48 10.1016/j.cmpb.2009.10.006 20036439

[B3] Yan Z Jiang Z Miyamoto A Wei Y The moment segmentation analysis of heart sound pattern Computer methods and programs in biomedicine 2010 98 2 140 150 10.1016/j.cmpb.2009.09.008 19854530

[B4] Dokur Z Olmez T Heart sound classification using wavelet transform and incremental self-organizing map Digital Signal Processing 2008 18 6 951 959 10.1016/j.dsp.2008.06.001

[B5] Olmez T Dokur Z Classification of heart sounds using an artificial neural network Pattern Recognition Letters 2003 24 1-3 617 629 10.1016/S0167-8655(02)00281-7

[B6] Gupta C Palaniappan R Swaminathan S Krishnan S Neural network classification of homomorphic segmented heart sounds Applied Soft Computing 2007 7 286 297 10.1016/j.asoc.2005.06.006

[B7] Ahlstrom C Lanne T Ask P Johansson A A method for accurate localization of the first heart sound and possible applications Physiological Measurement 2008 29 417 10.1088/0967-3334/29/3/011 18367815

[B8] Arvin F Doraisamy S Real-time segmentation of heart sound pattern with amplitude reconstruction IEEE EMBS Conference on Biomedical Engineering and Sciences 2010 130 133

[B9] Bello J Sandler M Blackboard system and top-down processing for the transcription of simple polyphonic music COST G-6 Conference on Digital Audio Effects (DAFx-01) 2001

[B10] Plumbley M Abdallah S Bello J Davies M Monti G Sandler M Automatic music transcription and audio source separation Cybernetics and Systems 2002 33 6 603 627 10.1080/01969720290040777

[B11] Ryynanen M Klapuri A Polyphonic music transcription using note event modeling Applications of Signal Processing to Audio and Acoustics, 2005. IEEE Workshop on, IEEE 2005 319 322

[B12] Arvin F Doraisamy S Real-Time Pitch Extraction of Acoustical Signals Using Windowing Approach Australian Journal of Basic and Applied Sciences 2009 3 4 3557 3563

[B13] Modegi T Iisaku S Proposals of MIDI coding and its application for audio authoring Multimedia Computing and Systems, 1998. Proceedings. IEEE International Conference on, IEEE 1998 305 314

[B14] Modegi T MIDI encoding method based on variable frame-length analysis and its evaluation of coding precision Multimedia and Expo, 2000. ICME 2000. 2000 IEEE International Conference on, IEEE 2000 2 1043 1046

[B15] Modegi T Studies in Health Technology and Informatics Studies in health technology and informatics 2001 84 366 370 11604765

[B16] Doraisamy S Polyphonic Music Retrieval: The N-gram Approach PhD thesis 2004 Department of Computing, Imperial College London

[B17] Doraisamy S Ruger S Robust polyphonic music retrieval with n-grams Journal of Intelligent Information Systems 2003 21 53 70 10.1023/A:1023553801115

[B18] Arvin F Doraisamy S Heart Sound Musical Transcription Technique Using Multi-Level Preparation International Review on Computers and Software 2010 5 6 595 600

[B19] Phua K Chen J Dat T Shue L Heart sound as a biometric Pattern Recognition 2008 41 3 906 919 10.1016/j.patcog.2007.07.018

[B20] Jiang Z Choi S A cardiac sound characteristic waveform method for in-home heart disorder monitoring with electric stethoscope Expert Systems with Applications 2006 31 2 286 298 10.1016/j.eswa.2005.09.025

[B21] Khorasani E Doraisamy S Arvin F An Approach for Heartbeat Sound Transcription International Conference on Computer Technology and Development, IEEE 2009 38 41

